# Linking species functional traits of terrestrial vertebrates and environmental filters: A case study in temperate mountain systems

**DOI:** 10.1371/journal.pone.0211760

**Published:** 2019-02-07

**Authors:** Paula García-Llamas, Thiago Fernando Rangel, Leonor Calvo, Susana Suárez-Seoane

**Affiliations:** 1 Biodiversity and Environmental Management Dpt., Faculty of Biological and Environmental Sciences, University of León, León, Spain; 2 Ecology Dpt., Institute of Biological Sciences, UFG, Goiânia, GO, Brazil; University of Georgia, UNITED STATES

## Abstract

Knowledge on the relationships between species functional traits and environmental filters is key to understanding the mechanisms underlying the current patterns of biodiversity loss from a multi-taxa perspective. The aim of this study was to identify the main environmental factors driving the functional structure of a terrestrial vertebrate community (mammals, breeding birds, reptiles and amphibians) in a temperate mountain system (the Cantabrian Mountains; NW Spain). Based on the Spanish Inventory of Terrestrial Vertebrate Species, we selected three functional traits (feeding guild, habitat use type and daily activity) and defined, for each trait, a set of functional groups considering vertebrate species with common functional characteristics. The community functional structure was evaluated by means of two functional indexes indicative of functional redundancy (species richness within each functional group) and functional diversity. Ordinary least squares regression and conditional autoregressive models were applied to determine the response of community functional structure to environmental filters (climate, topography, land cover, physiological state of vegetation, landscape heterogeneity and human influence). The results revealed that both functional redundancy and diversity of terrestrial vertebrates were non-randomly distributed across space; rather, they were driven by environmental filters. Climate, topography and human influence were the best predictors of community functional structure. The influence of land cover, physiological state of vegetation and landscape heterogeneity varied among functional groups. The results of this study are useful to identify the general assembly rules of species functional traits and to illustrate the importance of environmental filters in determining functional structure of terrestrial vertebrate communities in mountain systems.

## Introduction

Mountains are highly valuable landscapes that hold a great proportion of the world’s biodiversity [1] and constitute important centers of endemism, endangered species and ecosystems [2]. They are highly fragile systems, especially susceptible to biodiversity loss due to their vulnerability to human and natural disturbances [3]. Biodiversity loss can be mainly attributed to climate and land use changes, as well as to species introduction and nitrogen deposition, which are major environmental problems impacting biological systems worldwide [4, 5, 6].

Biodiversity declines may affect the processes and functioning of ecosystems [7] and, ultimately, compromise the capacity of ecological systems to provide goods and services that support human well-being [8]. Therefore, the quantification of biodiversity loss is of utmost importance within the framework of conservation strategies [9]. Biodiversity estimation has most often focused on taxonomic species richness while, in comparison, other components of biodiversity (e.g., genetic diversity, functional diversity and beta diversity) have been under-evaluated [10]. Species Functional Traits (SFT) can be defined as any morphological, biochemical, physiological, structural, phenological or behavioral attribute of an organism that influences fitness, their responses to the environment and ecosystem processes [11, 12]. Ecological processes are generally mainly ruled by functional attributes of the organisms rather than by their taxonomic status, as different taxa may be functionally similar [13]. The evaluation of species functional traits will thus contribute to a greater understanding of such ecological processes, as well as the potential resilience of ecosystems to environmental change [4]. Consequently, trait-based approaches tackling functional diversity have recently attracted growing interest amongst the scientific community [14, 15].

The effects of biodiversity on ecological processes and functioning depend on the ecological differences among the species in the community [16]. The ecological requirements of organisms largely determine the response of the regional species pool to environmental filters [17] and, thus, the assembly of SFT at any particular site [18]. Environmental filters are non-random ecological factors that may restrict or exclude species with unviable physiological limits from coexisting in or entering a community [19]. SFT are not filtered independently from each other, but usually associate in patterns that enable the classification of the species pool into a few functional types with similar environmental responses and influences on ecosystem processes [20]. According to Diaz et al. [21], climate, disturbance regimes and landscape heterogeneity are major environmental filters operating to restrict or exclude SFT at any specific site. For example, altitudinal variations in temperature and summer drought have been demonstrated to affect the SFT of plants in Mediterranean mountains [22]. Similarly, temperature gradients may constrain the functional response of animal species, such as attack rate and maximal intake rate [23]. Furthermore, landscape heterogeneity may affect the range of SFT in bird communities due to species habitat requirements [24]. However, the role of these environmental filters can be modified by global change, leading to non-random biodiversity loss and functional shifting [21]. Thus, understanding the SFT-environment relationship is of great importance, not only for determining the spatial distribution of SFT, but also for assessing biodiversity loss patterns under different scenarios of global change [25]. In particular, mountains provide suitable scenarios to evaluate the role of environmental filters on SFT, because strong environmental gradients (i.e., temperature and precipitation) associated with elevation usually constrain the distribution of plants and animals [26].

Several authors have highlighted the need for multi-taxa approaches [27, 28] based on grouping similar functional responses across unrelated taxa, to evaluate the responses of SFT against environmental filtering. These approaches match the ecological concept of functional convergence that entails a similar adaptive response of species to environmental factors [29]. They will thus enable the generalization of results and the establishment of global patterns of species’ response to the environment, and therefore aid in conservation management [30]. Nevertheless, most recent functional studies in the literature have been restricted to specific taxonomic groups, mainly plants, invertebrates and birds [31, 32]. This is probably due to: (i) the availability of free accessible trait databases for these groups [33] and (ii) the challenge of dealing with other taxonomic groups with high variability in behavior, morphology and foraging strategies [34].

In this study, we developed a multi-taxa approach to analyze SFT-environment relationships across terrestrial vertebrates (mammals, breeding birds, reptiles and amphibians) and to assess the role of environmental filters in structuring SFT assembly, using a temperate mountain system (the Cantabrian Mountains, NW Spain) as a case study. In particular, our specific goals were to identify: (i) major groups of environmental variables that govern community functional structure of terrestrial vertebrate species; and (ii) common patterns of response of functional groups to environmental filters.

## Methods

### Study area

**Fig 1:**
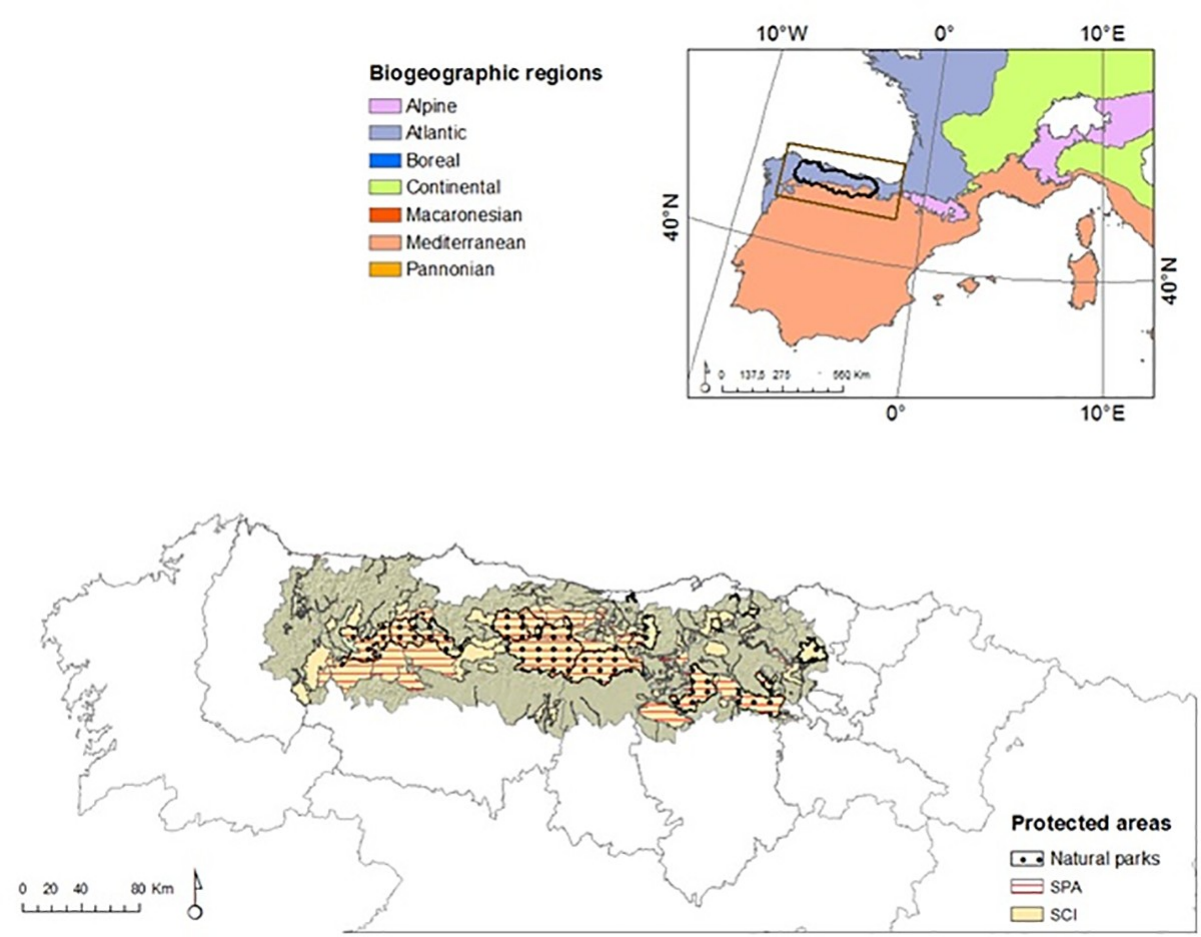
Study area: The Cantabrian Mountains located in NW spain. Information on biogeographic regions was obtained from the Spanish Ministry of Agriculture, Food and Environment (http://www.magrama.gob.es/). Information on protected areas was obtained from the BCN200 database of the Spanish Geographic Institute (www.ign.es); SPC Special Protection Areas, SCI Sites of Community Importance. Figure was created in ArcGIS 10.2 (Esri 2014).

The Cantabrian Mountains (NW of Spain) cover approximately 31,494 km^2^, with an altitude ranging from sea level to 2631 m.a.s.l. (Fig 1). They lie at the limit between the Eurosiberian (northern slope) and Mediterranean (southern slope) biogeographic regions [35]. The climate changes from Temperate-Oceanic to Mediterranean between the two slopes [35]. These particular climatic characteristics, the uneven topography and the historical land management, based on burning, cutting and grazing, have resulted in a very heterogeneous landscape mosaic of special relevance from a conservation perspective [36], hosting a wide variety of ecosystems, habitats and endemic species. Moreover, the Cantabrian Mountains are partially included within the Mediterranean basin, which is recognized as a biodiversity hotspot [37]. Because of this conservation importance, around 40% of the surface area is under some category of protection. The landscape is dominated by pastures and croplands in valley bottoms and lowlands, changing in the mid-high slopes to heathlands, shrublands and deciduous forests dominated by *Fagus sylvatica*, *Betula pubescens*, *Quercus petraea* and *Q*. *robur*, on northern slopes, and *Q*. *pyrenaica* and *Q*. *rotundifolia* on southern slopes. Plantations of *Pinus pinaster*, *P*. *radiata* and *Eucalyptus globulus* also cover middle slopes, replacing scrublands and heathlands. The top of the mountains is covered by natural grasslands and rock formations [38].

### Species functional traits, functional groups and functional indices of vertebrate species

Species functional traits (SFT) were selected according to the criteria of Chillo & Ojeda [39] and Grave et al. [40], and included resource capture (feeding guild) and behavior (habitat use type and daily activity) traits, that can be seen as drivers of biodiversity and ecosystem function relationships [41]. Feeding guild is related to the resource requirements of species in the community [42], while habitat use type and daily activity are associated with the spatial distribution and temporal use of resources [27]. For each of these three traits, we selected a range of functional groups of terrestrial vertebrate species (mammals, breeding birds, reptiles and amphibians; Table 1) that shared common functional characteristics. This implies a similar functional adaptive response to environmental factors across taxa, which enables comparability.

**Table 1:** Species functional traits and functional groups considered in this study.

Trait	Range of functional groups
Feeding guild	Carnivore, granivore, herbivore, omnivore, insectivore
Habitat use type	Tree-dwelling, terrestrial, ground-dwelling, cave-dwelling, rock-dwelling, semi-aquatic, shrub-dwelling, anthropogenic environments, generalist
Daily activity	Nocturnal, diurnal, multiphasic

Terrestrial vertebrate functional groups were identified based on experts’ knowledge using the official database of vertebrates of Spain (Ministry of Agriculture, Food and Environment 2012; www.magrama.gob.es; see S1 Table). This database contains information on species occurrence (presence/absence) in a 10x10 km UTM grid system for the period 1980–2007. Data were collected from published sources and field surveys carried out by volunteers through direct and indirect observations (pellets, tracks or bed sites) [43, 44, 45].

The community functional structure was characterized by means of two functional indexes accounting for functional redundancy and diversity. These were calculated for each of the 388 UTM 10x10 km grid squares covering the study area. We estimated functional redundancy as the richness (total number of species) within each functional group [10]. Further, we quantified functional diversity via the Petchey and Gaston functional diversity index (hereafter–FD) [46, 47]. This is a continuous measure of functional diversity, which is based on the sum of the total branch length of a dendrogram obtained from distance matrixes. Gower’s distance (a metric that can handle ratio, nominal and interval data) [48] and the unweighted pair-group clustering method with arithmetic averages (UPGMA) were used to obtain the distance matrix and the functional dendrogram. This functional diversity index does not require abundance data, allows the assessment of multiple functional traits and has desirable statistical properties (i.e., the addition of a species will not decrease the functional diversity of the community) [47].

### Environmental filters

A set of 43 environmental variables accounting for climate, topography, land cover, physiological state of vegetation, landscape heterogeneity, human influence and accessibility were chosen as environmental filters (S2 Table) [49, 50, 51].

Climatic variables are expected to define significant variations in species distribution at a regional scale [17]. The maximum and minimum temperature, as well as the mean precipitation, were obtained on a monthly basis (period 1951–1999) from the Ninyerola’s Climatic Atlas [52] at a 200 m spatial resolution. These values were averaged over summer (July, August and September) and winter (December, January and February) seasons, because these periods are the most limiting seasons for Temperate and Mediterranean species [53, 54]. We calculated the mean and the standard deviation of the seasonal climatic variables (as an expression of the general climatic pattern and the climatic variability pattern, respectively) for each 10x10 km UTM square, in correspondence with the grid reference system of the vertebrate species inventory.

Topography influences land cover characteristics, microclimatic conditions, as well as species movement, iterations [55] and visual communication [56]. Topographic variables (altitude, slope and solar radiation) were derived from a Digital Elevation Model (DEM) at 90 m resolution obtained from the Spanish Geographic Institute (www.ign.es). The mean and the standard deviation of each topographic variable were calculated for each 10x10 km UTM square.

Land cover is known to influence species habitat selection, as it reflects resource quality and availability [57]. Land cover information was obtained from the CORINE Land Cover database for the year 2006 at 30 m resolution (http://www.eea.europa.eu/publications/COR0-landcover). A reclassification of the original CORINE dataset (44 classes) was carried out considering the vertical structure of vegetation, with the purpose of simplifying the original dataset. This resulted in a new land cover database comprising 12 classes with an accuracy of 82.5% (see more details in S3 Table and [38]). The relative frequency of each land cover class was subsequently calculated for each 10x10 km UTM square.

The Normalized Difference Vegetation Index (NDVI) was used as an indicator of the physiological state of vegetation. It varies from -1 (in non-vegetated areas) to +1 (indicating increasing vegetation greenness) [58]. This index has been broadly recognized as a driver of species distribution-resource availability relationships [51]. The annual mean value of NDVI was derived from a temporal monthly series of NDVI based on the following years: 1983, 1985, 1990, 1993, 1996 and 1999, obtained from NOAA-AVHRR at 1 km resolution (see [59] for technical details).

This NDVI database was also used to calculate landscape heterogeneity (see [60] for similar approaches) as an indicator of habitat availability. Landscape heterogeneity can give rise to large spatial variations in reflectance and, consequently, in NDVI spatial patterns [61]. NDVI values were divided into 20 classes according to data distribution in a frequency histogram. Landscape heterogeneity was estimated as the number of NDVI classes (i.e. richness of NDVI classes) in each 10x10 km UTM square.

Human influence variables can reflect the degree of anthropogenic disturbances [56]. As indicators, we used the minimum Euclidean distance from each pixel to urban settlements (mean and standard deviation values at each 10x10 km UTM square), the surface of protected areas (in km^2^) and the protection status (i.e., presence/absence of protected areas at each pixel).

A major concern of using species data derived from surveys based on direct observations is related to differences in detectability between habitats or species [57]. Therefore, in order to account for potential sampling bias in species surveys, a variable indicating the accessibility of each pixel and thus the potential cost of surveying, was included in the analysis, although it was not considered as an environmental filter. We obtained the mean cost of accessibility for each 10x10 km UTM square from a map at 90 m resolution that integrated data on slope, Euclidean distance from each pixel to paths, and Euclidean distance from each pixel to settlements. We also estimated the cost of accessibility as the total length of paths and roads.

Information on settlements, roads, paths and protected areas was obtained at 1:200000 spatial resolution, from the BCN200 database of the Spanish Geographic Institute (www.ign.es). The slope was derived from a DEMs at 90 m resolution (www.ign.es).

### Data analysis

To explore the response of functional redundancy and functional diversity to environmental filters, we fitted separated multi-regression linear models (ordinary least squares; OLS). Before running these models, we carried out exploratory data analysis to detect multicollinearity problems, through the evaluation of Spearman’s bivariate correlations among all environmental predictors. The threshold of 0.70 (*r*^*2*^ > 0.7) was used as the criteria for identifying pairs of correlated variables. From each pair, the variable with the least ecological meaning was removed from subsequent analyses [56]. Thus, the original pool of environmental variables (S2 Table) was simplified to 28 variables, which were entered as predictors in the OLS models (Table 2). Additionally, we calculated the variance inflation factor (VIF), removing those predictors that achieved a value higher than 5 in the OLS models [62].

**Table 2:** Environmental variables entered as predictors in OLS models after excluding correlated variables. The mean and/or the standard deviation value of the environmental variables were extracted for each 10x10 km UTM sampling unit (See S2 Table for more details).

Family	Code	Description of the variable	Source
Climate	PRECWIN	Mean precipitation (mm) in winter	Ninyerola’s Climatic Atlas [48] at 200 m spatial resolution
	TMAXWIN	Maximum temperature (°C) in winter	
	TMAXSUM	Maximum temperature (°C) in summer	
	stdPRECWIN	Standard deviation of mean precipitation (mm) in winter	
	stdTMAXSUM	Standard deviation of maximum temperature (°C) in summer	
Topography	SOLR	Solar radiation (*10^6^ W/h)	Digital Elevation Model at 90 m spatial resolution from the Spanish Geographic Institute
	stdDEM	Standard deviation of elevation (m)	
	stdSLO	Standard deviation of slope (%)	
Land cover	INFRA	Frequency of human infrastructures	CORINE Land Cover 2006 at 30 m spatial resolution
	MIN	Frequency of mineral extraction sites	
	HERC	Frequency of herbaceous croplands	
	WOOC	Frequency of woody cropland	
	PAS	Frequency of pasturelands	
	FOR	Frequency of forest	
	TWOOD	Frequency of transitional woodland-shrublands	
	SCRUB	Frequency of scrub and sclerophyllous-herbaceous formations	
	SPAR	Frequency of sparsely vegetated areas	
	BARE	Frequency of bare areas	
	WET	Frequency of wetlands	
	WAT	Frequency of water	
Physiological state of vegetation	NDVI	Annual average NDVI index	NDVI from NOAA-AVHRR at 1 km spatial resolution
Landscape heterogeneity	LANDHET	Landscape heterogeneity computed as richness of NDVI classes	NDVI from NOAA-AVHRR at 1 km spatial resolution
Human influence	UD	Euclidean distance to the nearest settlement (m)	Vector layers at 1:200000 spatial resolution from the Spanish Geographic Institute
	stdUD	Standard deviation of Euclidean distance to the nearest settlement (m)	
	SURFPA	Surface covered by protected areas in each sampling unit (km^2^)	
	PREPA	Presence/absence of protected areas	
Accessibility	LROAD	Total length of roads and paths (km)	Vector layers of roads at 1:200000 spatial resolution
	ACOST	Accessibility cost at 90m spatial resolution	Digital Elevation Models at 90 m spatial resolution and vector layers of roads and settlements at 1:200000 spatial resolution, from the Spanish Geographic Institute

A stepwise procedure (backward and forward variable selection) according to Akaike’s information criterion (AIC) was used to test the effect of all predictors on the response variables and select the best-fit model. The residuals of OLS models were graphically checked for normal distribution, independence and homoscedasticity. Spatial autocorrelation in the residuals, which violates the assumption of independence in their distribution, was further evaluated using the Moran’s index. When evidence of spatial autocorrelation was detected (Moran’s index > 0.1), we applied Simultaneous or Conditional Autoregressive (SAR and CAR, respectively) models [63].

All statistical analyses were performed using SAM v4.0 statistical software [64]. Environmental variables were processed using ArcGIS 10.2 (Esri 2014).

## Results

**Fig 2:**
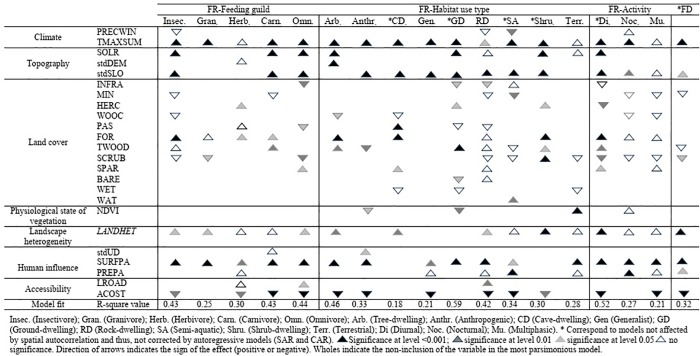
Results of the most parsimonious models (ordinary least squares regression and autoregressive models SAR or CAR) testing the effect of environmental predictors on both functional redundancy (FR) and diversity (FD). *S*ignificance levels, sign of the effect and variance explained by models are indicated. See Table 2 for codes of environmental variables. Only variables included as predictors in some of the most parsimonious models are shown.

Regarding functional redundancy, models of feeding guild SFT achieved R^2^ values between 0.25 (granivores) and 0.44 (omnivores). Considering models of habitat use SFT, values of R^2^ ranged from 0.18 (cave-dwelling group) to 0.59 (ground-dwelling group). Models of daily activity SFT achieved R^2^ values between 0.21 (multiphasic functional group) and 0.52 (diurnal group). The functional diversity model obtained a R^2^ value of 0.32 (Fig 2).

As a general trend, both functional redundancy and diversity were mainly explained by climate, topography, human influence and accessibility. Specifically, maximum summer temperature, standard deviation of the slope and surface of protected areas systematically emerged as the primary variables influencing community functional structure. Land cover, physiological state of vegetation and landscape heterogeneity were the weakest predictors (Fig 2 and S4 Table).

Additionally, solar radiation substantially influenced the functional redundancy of the considered SFTs, but had no effect on functional diversity. In particular, this environmental filter significantly affected the following functional groups: insectivores, carnivores and omnivores (feeding guild SFT); tree-dwellers, ground-dwellers and shrub-dwellers (habitat use SFT); and diurnals (activity SFT). Moreover, land cover classes had a substantial influence on the functional redundancy of only some functional groups, as well as on functional diversity. The richness of generalist, terrestrial, multiphasic and nocturnal groups was not significantly related to any land cover class. Nevertheless, the richness of insectivores, herbivores, carnivores, tree-dwellers, cave-dwellers, ground-dwellers, shrub-dwellers and diurnals increased with the presence of woody land cover, while the richness of the anthropogenic group decreased. Herbivores, cave-dwellers, ground-dwellers and shrub-dwellers were associated with open areas, such as herbaceous croplands and pasturelands. However, the presence of agricultural areas negatively affected the richness of omnivores, tree-dwellers and diurnals. Human infrastructures hampered the richness of omnivores, ground-dwellers, rock-dwellers and diurnals. Ground-dwellers were also negatively affected by bare areas. The functional redundancy and functional diversity index of granivores, omnivores and diurnals were disfavored by scrublands and sclerophyllous-herbaceous vegetation. Additionally, the richness of semi-aquatic species was positively influenced by water bodies (Fig 2 and S4 Table).

The cost of accessibility was negatively related to functional redundancy and diversity. Road length also showed significant relationships with the richness of herbivores, omnivores and rock-dwellers (Fig 2 and S4 Table).

## Discussion

This study showed that both functional redundancy and diversity were non-randomly distributed across the Cantabrian Mountains and, consequently, environmental filters structured the species functional traits’ assemblages. Climate emerged as a main environmental filter of SFTs, whose effects might respond to the strategic geographic position of the Cantabrian Mountains, located in a transitional area under the influence of Mediterranean and Temperate-Oceanic climates [35]. In areas of Mediterranean influence, maximum summer temperature usually constitutes a seasonally limiting factor for species, due to its effect on food and water flows [53]. Meanwhile, Hawkins et al. [65] noted that low temperatures are critical for species occurrence in Temperate areas, and supported the idea of greater species richness at higher temperatures. In this context, the study area, with hot summers and a dry summer period of less than two months [66], might partially meet the ecological requirements of both Mediterranean and Temperate species. This could explain the co-occurrence of functional groups and thus, the high values of functional diversity and redundancy. These results are consistent with previous studies showing the importance of transition areas for preserving functional diversity [67]. Moreover, according to the energy hypothesis, the energy available in the system is a limiting factor for biodiversity, so that more species would tend to coexist in areas of high energy availability [65]. This may further explain the role of temperature and solar radiation in terrestrial vertebrate community functional structure, as these two variables are highly correlated with the energy supply in the environment [58, 68].

Functional redundancy and diversity were also positively associated with the standard deviation of the slope. The importance of topography for structuring species assembly in mountains has been detected in other systems, such as tropical forests [69]. Slope is generally related to terrain roughness, which can affect the energetic and timely cost of species’ movements and, hence, the use of resources by species [55]. Accordingly, slope variability may favor the presence of species with different functional attributes through the use of complementary resources, which would increase functional diversity [21]. This could be explained by the fact that slope variability enables species to exploit different habitats according to their dispersal and movement abilities. Furthermore, the increase in functional redundancy with slope variability may contribute to maintaining the properties of ecosystems, since greater numbers of functionally similar species (i.e. functional redundancy) increases the probability that some species will overcome perturbations or changes in the system [20].

Functional redundancy and diversity were weakly explained by land cover, with the role of land cover in structuring SFT strongly dependent on the particular ecological requirements of species. For example, functional groups with less specialized habitat requirements (i.e. generalists, terrestrials or multiphasics) did not show, overall, significant responses to land cover. This could explain the poor performance of the models built for these groups, as also reported by other studies [70]. Meanwhile, the weak association of granivores with land cover could be linked to their foraging strategy [71]. This strategy may involve different habitat requirements according to seed preferences, distribution or detectability, as well as toxic minimization, predation risk, competitors [72] or seasonal food availability [73, 74]. Conversely, carnivores and herbivorous were likely to occur in landscapes dominated by forests with open patches and grazing areas, since such heterogeneous landscape mosaics support a favorable combination of refuge and foraging provision [25, 75]. Likewise, tree-dwelling species were primarily associated with the tree canopy. In contrast, the negative effect of woody vegetation on species from anthropogenic environments could be related to the simplification of the vertical structure of vegetation in these ecosystems [76].

Landscape heterogeneity is expected to be a relevant environmental filter structuring SFTs within the community [24, 40]. In this context, Lee and Martin [77] stated that functional diversity is limited by the accessibility of ecological niches. Heterogeneous landscapes offer more niches and complementary resources [78] than homogeneous landscapes and, hence, more functional groups are expected to coexist in such heterogeneous areas. In the Cantabrian Mountains, we found a positive relationship between landscape heterogeneity and functional diversity, consistent with the findings of other studies carried out in Central America [79] and Romania [80]. In mountains, the landscape is heterogeneous [37] as a result of topographic and climatic complexity, as well as traditional human intervention. Nevertheless, in our study, functional redundancy was weakly explained by landscape heterogeneity, likely because not all SFT respond equally to this environmental filter [27]. Other types of functional traits, such as those related to dispersal capacity, body size or capacity of colonization, could probably be more related to landscape heterogeneity [25, 28]. Consequently, we suggest future research including these traits for a deeper understanding of the role played by landscape heterogeneity as a limiting factor for the functional response of species.

Anthropogenic disturbances may act as a major environmental filter by: (i) excluding species whose physiological tolerance is exceeded or whose habitat requirements are stable; or (ii) enabling the entry of new species according to their functional attributes (e.g. generalist species; [4]). Such filters usually lead to non-random functional simplifications of communities, with important implications for ecosystem processes [81]. In this way, protected areas are subject to the regulation of human activities leading to fewer threats (e.g. disturbances) and improving habitat quality [82], likely enabling the preservation of functional diversity and redundancy. For instance, the presence of carnivores usually presents a conflict with human interests, such as competition for resources or livestock predation. Low human pressure in protected areas may benefit carnivores in terms of conservation, habitat quality and human conflict limitation [83]. Coughenour [55] and Marchand et al. [84] also noted the positive contribution of protected areas on herbivore populations due to lower hunting pressure. In the Cantabrian Mountains, functional redundancy and diversity were positively correlated with land protection status, in contrast with other studies carried out in France [24] and the Iberian Peninsula [85]. These differences could be associated with the establishment of protected areas that are: (i) traditionally biased towards the protection of either specific taxonomic groups or taxonomic diversity [24]; and (ii) located around particular valuable and non-impacted systems, such as mountains, with lowlands remaining underrepresented [85].

The significant association of functional redundancy and diversity with the cost of accessibility suggests some degree of bias and gaps in species surveys, which is a relevant problem in many of the available species databases [86]. Differences in detectability of animals or signs, but also observers’ behavior, are the main constraints in direct presence/absence observation methods [57]. Nevertheless, such observation methods avoid uncertainties related to predictive models, like problems of independence among samples or arbitrariness in the selection of the study areas [87]. Consequently, despite limitations, they have been widely used in biodiversity studies [88].

## Conclusions

This study highlights the role played by climate, topography and human influence variables as main environmental filters determining SFT assembly patterns and functional structure of terrestrial vertebrate species in mountain systems. Our results indicated that landscape homogenization, occurring in a context of land use and land cover change, undermines functional diversity and, therefore, hinders ecosystem functioning in mountains. This study helps identify general rules driving species functional trait assemblages and illustrates the importance of environmental filters in determining the functional structure of terrestrial vertebrate communities in mountain systems. Finally, it stresses the need to develop functional approaches based on multi-taxa perspectives, for environmental management and conservation applications, in a context of environmental change.

## Supporting information

S1 Table List of species of mammals, breeding birds, reptiles and amphibians included in the official database of vertebrates of Spain (Ministry of Agriculture, Food and Environment 2012; www.magrama.gob.es), from which we derived the species functional groups. (DOCX)

S2 Table Environmental variables used as predictors. The average value and/or the standard deviation value (*std*) of the environmental variables was extracted at each UTM 10x10 km sampling unit.
(DOCX)

S3 Table CORINE land cover classes present in the Cantabrian Mountains. (DOCX)

S4 Table Results of the most parsimonious models (ordinary least squares regression and autoregresive models SAR or CAR) testing the effect of environmental predictors on the richness of each functional group, including the sign of the effect and standarized coefficient estimates of distinct predictors. Significant predictors appear in bold. See Table 2 for codes of environmental variables. Only variables included as predictors in the most parsimonious models are shown.
(DOCX)
