# Supplementary material for: Linking species functional traits of terrestrial vertebrates and environmental filters: A case study in temperate mountain systems
Source: PLoS One. 2019 Feb 7;14(2):e0211760. doi: 10.1371/journal.pone.0211760 (PMC6366930; doi:10.1371/journal.pone.0211760)
Supplement: S1 Table — (DOCX) [file pone.0211760.s001.docx]

**Supporting Information**

**Linking species functional traits of terrestrial vertebrates and environmental filters: a case study in temperate mountain systems**

Paula García-Llamas^1^, Thiago Fernando Rangel^2^, Leonor Calvo^1^**,** Susana Suárez-Seoane^1^

**S1 Table. List of species of mammals, breeding birds, reptiles and amphibians included in the official database of vertebrates of Spain (Ministry of Agriculture, Food and Environment 2012;** [**www.magrama.gob.es**](http://www.magrama.gob.es)**), from which we derived species functional groups**

| Species of mammals | | | |
| --- | --- | --- | --- |
| *Apodemus flavicollis* | *Hypsugo savii* | *Myocastor coypu* | *Pipistrellus pygmaeus* |
| *Apodemus sylvaticus* | *Lepus castroviejoi* | *Myodes glareolus* | *Plecotus auritus* |
| *Arvicola sapidus* | *Lepus europaeus* | *Myotis becnsteinii* | *Plecotus austriacus* |
| *Arvicola terrestris* | *Lepus granatensis* | *Myotis blythii* | *Rattus norvegicus* |
| *Barbastella barbastellus* | *Lutra lutra* | *Myotis daubentonii* | *Rattus rattus* |
| *Canis lupus* | *Martes foina* | *Myotis emarginatus* | *Rhinolophus euryale* |
| *Capra pyrenaica* | *Martes martes* | *Myotis myotis* | *Rhinolophus ferrumequinu* |
| *Capreolus capreolus* | *Meles meles* | *Myotis mystacinus* | *Rhinolophus hipposideros* |
| *Cervus elaphus* | *Micromys minutus* | *Myotis nattereri* | *Rhinolophus mehelyi* |
| *Chionomys nivalis* | *Microtus agrestis* | *Neomys anomalus* | *Rupicapra pyrenaica* |
| *Crocidura russula* | *Microtus arvalis* | *Neomys fodiens* | *Sciurus vulgaris* |
| *Crocidura suaveolens* | *Microtus duodecimcostatu* | *Neovison vison* | *Sorex coronatus* |
| *Dama dama* | *Microtus gerbei* | *Nyctalus lasiopterus* | *Sorex granarius* |
| *Eliomys quercinus* | *Microtus lusitanicus* | *Nyctalus leisleri* | *Sorex minutus* |
| *Eptesicus serotinus* | *Mus musculus* | *Nyctalus noctula* | *Suncus etruscus* |
| *Felis silvestris* | *Mus spretus* | *Oryctolagus cuniculus* | *Tadaria teniotis* |
| *Galemys pyrenaicus* | *Mustela erminea* | *Ovies aries* | *Talpa europaea* |
| *Genetta genetta* | *Mustela lutreola* | *Pipistrellus kuhlii* | *Talpa occidentalis* |
| *Glis glis* | *Mustela nivalis* | *Pipistrellus nathusii* | *Ursus arctos* |
| *Herpestes ichneumon* | *Mustela putorius* | *Pipistrellus pipistrellu* | *Vulpes vulpes* |

**S1 Table (cont.).**

| Species of breeding birds | | | |
| --- | --- | --- | --- |
| *Accipiter gentilis* | *Aythya ferina* | *Cisticola juncidis* | *Emberiza hortulana* |
| *Accipiter nisus* | *Aythya fuligula* | *Clamator glandarius* | *Emberiza schoeniclus* |
| *Acrocephalus arundinaceus* | *Bubo bubo* | *Coccothraustes coccothraustes* | *Erithacus rubecula* |
| *Acrocephalus scirpaceus* | *Bubulcus ibis* | *Columba domestica* | *Falco naumanni* |
| *Actitis hypoleucos* | *Burhinus oedicnemus* | *Columba livia/domestica* | *Falco peregrinus* |
| *Aegithalos caudatus* | *Buteo buteo* | *Columba oenas* | *Falco subbuteo* |
| *Alauda arvensis* | *Calandrella brachydactyla* | *Columba palumbus* | *Falco tinnunculus* |
| *Alcedo atthis* | *Caprimulgus europaeus* | *Coracias garrulus* | *Ficedula hypoleuca* |
| *Alectoris rufa* | *Carduelis cannabina* | *Corvus corax* | *Fringilla coelebs* |
| *Anas acuta* | *Carduelis carduelis* | *Corvus corone* | *Fulica atra* |
| *Anas clypeata* | *Carduelis chloris* | *Corvus frugilegus* | *Galerida cristata* |
| *Anas crecca* | *Carduelis spinus* | *Corvus monedula* | *Galerida theklae* |
| *Anas platyrhynchos* | *Cecropis daurica* | *Coturnix coturnix* | *Gallinula chloropus* |
| *Anas strepera* | *Certhia brachydactyla* | *Cuculus canorus* | *Garrulus glandarius* |
| *Anthus campestris* | *Certhia familiaris* | *Delichon urbicum* | *Gyps fulvus* |
| *Anthus pratensis* | *Cettia cetti* | *Dendrocopos major* | *Haematopus ostralegus* |
| *Anthus spinoletta* | *Charadrius alexandrinus* | *Dendrocopos medius* | *Hieraaetus fasciatus* |
| *Anthus trivialis* | *Charadrius dubius* | *Dendrocopos minor* | *Hieraaetus pennatus* |
| *Apus apus* | *Chersophilus duponti* | *Dryocopus martius* | *Hippolais polyglotta* |
| *Apus melba* | *Ciconia ciconia* | *Egretta garzetta* | *Hirundo rustica* |
| *Aquila chrysaetos* | *Cinclus cinclus* | *Elanus caeruleus* | *Hydrobates pelagicus* |
| *Ardea cinerea* | *Circaetus gallicus* | *Emberiza calandra* | *Ixobrychus minutus* |
| *Asio flammeus* | *Circus aeruginosus* | *Emberiza cia* | *Jynx torquilla* |
| *Asio otus* | *Circus cyaneus* | *Emberiza cirlus* | *Lanius collurio* |
| *Athene noctua* | *Circus pygargus* | *Emberiza citrinella* | *Lanius excubitor* |
| *Accipiter gentilis* | *Aythya ferina* | *Cisticola juncidis* | *Emberiza hortulana* |
| *Accipiter nisus* | *Aythya fuligula* | *Clamator glandarius* | *Emberiza schoeniclus* |

**S1 Table (cont.).**

| Species of breeding birds | | | |
| --- | --- | --- | --- |
| *Lanius senator* | *Larus fuscus* | *Otis tarda* | *Podiceps cristatus* |
| *Larus fuscus* | *Otis tarda* | *Otus scops* | *Podiceps nigricollis* |
| *Larus michahellis* | *Otus scops* | *Podiceps cristatus* | *Strix aluco* |
| *Locustella naevia* | *Parus ater* | *Podiceps nigricollis* | *Sturnus unicolor* |
| *Loxia curvirostra* | *Parus caeruleus* | *Porzana porzana* | *Sturnus vulgaris* |
| *Lullula arborea* | *Parus cristatus* | *Porzana pusilla* | *Sylvia atricapilla* |
| *Luscinia megarhynchos* | *Parus major* | *Prunella collaris* | *Sylvia borin* |
| *Luscinia svecica* | *Parus palustris* | *Prunella modularis* | *Sylvia cantillans* |
| *Melanocorypha calandra* | *Passer domesticus* | *Pterocles orientalis* | *Sylvia communis* |
| *Merops apiaster* | *Passer hispaniolensis* | *Ptyonoprogne rupestris* | *Sylvia conspicillata* |
| *Milvus migrans* | *Passer montanus* | *Pyrrhocorax graculus* | *Sylvia hortensis* |
| *Milvus milvus* | *Perdix perdix* | *Pyrrhocorax pyrrhocorax* | *Sylvia melanocephala* |
| *Monticola saxatilis* | *Pernis apivorus* | *Pyrrhula pyrrhula* | *Sylvia undata* |
| *Monticola solitarius* | *Petronia petronia* | *Rallus aquaticus* | *Tachybaptus ruficollis* |
| *Montifringilla nivalis* | *Phalacrocorax aristotelis* | *Regulus ignicapilla* | *Tetrao urogallus* |
| *Motacilla alba* | *Phalacrocorax carbo* | *Regulus regulus* | *Tetrax tetrax* |
| *Motacilla cinerea* | *Phasianus colchicus* | *Remiz pendulinus* | *Tichodroma muraria* |
| *Motacilla flava* | *Phoenicurus ochruros* | *Riparia riparia* | *Tringa totanus* |
| *Muscicapa striata* | *Phoenicurus phoenicurus* | *Saxicola rubetra* | *Troglodytes troglodytes* |
| *Neophron percnopterus* | *Phylloscopus bonelli* | *Saxicola torquatus* | *Turdus merula* |
| *Numenius arquata* | *Phylloscopus collybita/ibericus* | *Scolopax rusticola* | *Turdus philomelos* |
| *Nycticorax nycticorax* | *Phylloscopus ibericus* | *Serinus citrinella* | *Turdus torquatus* |
| *Oenanthe hispanica* | *Phylloscopus sibilatrix* | *Serinus serinus* | *Turdus viscivorus* |
| *Oenanthe oenanthe* | *Phylloscopus trochilus* | *Sitta europaea* | *Tyto alba* |
| *Oriolus oriolus* | *Pica pica* | *Streptopelia decaocto* | *Upupa epops* |
| *Lanius senator* | *Picus viridis* | *Streptopelia turtur* | *Vanellus vanellus* |

**S1 Table (cont.).**

| Species of reptiles | | | |
| --- | --- | --- | --- |
| *Anguis fragilis* | *Eretmochelys imbricata* | *Mauremys leprosa* | *Psammodromus hispanicus* |
| *Chalcides bedriagai* | *Iberolacerta monticola* | *Natrix maura* | *Rhinechis scalaris* |
| *Chalcides striatus* | *Lacerta bilineata* | *Natrix natrix* | *Tarentola mauritanica* |
| *Coronella austriaca* | *Lacerta lepida* | *Podarcis bocagei* | *Trachemys scripta* |
| *Coronella girondica* | *Lacerta schreiberi* | *Podarcis hispanica* | *Vipera aspis* |
| *Dermochelys coriacea* | *Lacerta vivipara* | *Podarcis muralis* | *Vipera latasti* |
| *Emys orbicularis* | *Malpolon monspessulanus* | *Psammodromus algirus* | *Vipera seoanei* |
| Species of amphibians | | | |
| *Alytes obstetricans* | *Discoglossus jeanneae* | *Pelobates cultripes* | *Rana perezi* |
| *Bufo bufo* | *Hyla arborea* | *Pelodytes punctatus* | *Rana temporaria* |
| *Bufo calamita* | *Lissotriton boscai* | *Pleurodeles waltl* | *Salamandra salamandra* |
| *Chioglossa lusitanica* | *Lissotriton helveticus* | *Rana dalmatina* | *Triturus marmoratus* |
| *Discoglossus galganoi* | *Mesotriton alpestris* | *Rana iberica* |  |
