# Supplementary material for: Linking species functional traits of terrestrial vertebrates and environmental filters: A case study in temperate mountain systems
Source: PLoS One. 2019 Feb 7;14(2):e0211760. doi: 10.1371/journal.pone.0211760 (PMC6366930; doi:10.1371/journal.pone.0211760)
Supplement: S2 Table — The average value and/or the standard deviation value (std) of the environmental variables was extracted at each UTM 10x10 km sampling unit. (DOCX) [file pone.0211760.s002.docx]

**Supporting Information**

**Linking species functional traits of terrestrial vertebrates and environmental filters: a case study in temperate mountain systems**

Paula García-Llamas^1^, Thiago Fernando Rangel^2^, Leonor Calvo^1^**,** Susana Suárez-Seoane^1^

**S2 Table. Environmental variables used as predictors.** The average value and/or the standard deviation value (*std*) of the environmental variables was extracted at each UTM 10x10 km sampling unit.

| Family | Code | Variables description | Source |
| --- | --- | --- | --- |
|  | PRECSUM | Mean precipitation in summer (mm) | Ninyerola’s Climatic Atlas (Ninyerola et al. 2005) at 200 m spatial resolution |
|  | PRECWIN | Mean precipitation in winter (mm) |  |
|  | TMWIN | Mean temperature in winter (ºC) |  |
|  | TMSUM | Mean temperature in summer (ºC) |  |
|  | TMAXWIN | Maximum temperature in winter (ºC) |  |
|  | TMAXSUM | Maximum temperature in summer (ºC) |  |
|  | TMINWIN | Minimum temperature in winter (ºC) |  |
|  | TMINSUM | Minimum temperature in summer (ºC) |  |
|  | stdPRECSUM | Standard deviation of mean precipitation in summer (mm) |  |
|  | stdPRECWIN | Standard deviation of mean precipitation in winter (mm) |  |
|  | stdTMWIN | Standard deviation of mean temperature in winter (ºC) |  |
|  | stdTMSUM | Standard deviation of mean temperature in summer (ºC) |  |
|  | stdTMAXWIN | Standard deviation of maximum temperature in winter (ºC) |  |
|  | stdTMAXSUM | Standard deviation of maximum temperature in summer (ºC) |  |
|  | stdTMINWIN | Standard deviation of minimum temperature in winter (ºC) |  |
|  | stdTMINSUM | Standard deviation of minimum temperature in summer (ºC) |  |
| Topography | DEM | Elevation (m) | Digital Elevation Model (DEM) at 90 m spatial resolution from the Spanish Geographic Institute ([www.ign.es](http://www.ign.es)) |
|  | SLO | Slope (%) |  |
|  | SOLR | Solar radiation (*10^6^ W/h) |  |
|  | stdDEM | Standard deviation of elevation (m) |  |
|  | stdSLO | Standard deviation of slope (%) |  |
|  | stdSOLR | Standard deviation of solar radiation (*10^6^ W/h) |  |

**S2 Table (cont.).**

| Family | Code | Description of the variable | Source |
| --- | --- | --- | --- |
| Land cover | INFRA | Frequency of class human infrastructures | CORINE Land Cover 2006 at 30 m spatial resolution (<http://www.eea.europa.eu/publications/COR0-landcover>) |
|  | MIN | Frequency of class mineral extraction sites |  |
|  | HERC | Frequency of class herbaceous croplands |  |
|  | WOOC | Frequency of class woody croplands |  |
|  | PAS | Frequency of class pasturelands |  |
|  | FOR | Frequency of class forest |  |
|  | TWOOD | Frequency of class transitional woodland-shrublands |  |
|  | SCRUB | Frequency of class scrub and sclerophyllous-herbaceous formations |  |
|  | SPAR | Frequency of class sparsely vegetated areas |  |
|  | BARE | Frequency of class bare areas |  |
|  | WET | Frequency of class wetlands |  |
|  | WAT | Frequency of class water |  |
| Physiological state of vegetation | NDVI | Annual mean NDVI index  (no units, ranging from -1 to +1) | NDVI from NOAA-AVHRR  at 1 km of spatial resolution |
| Landscape heterogeneity | LANDHET | Landscape heterogeneity measured as the number of landscape classes (considering the total number of pixels in each sampling unit) | NDVI from NOAA-AVHRR  at 1 km of spatial resolution |
| Human influence | UD | Distance to the nearest settlement measured as Euclidian distance (m) | Vector layers at 1:200000 spatial resolution from the Spanish Geographic Institute ([www.ign.es](http://www.ign.es)) |
|  | stdUD | Standard deviation of the distance to the nearest settlement (m) |  |
|  | SURFPA | Surface of sampling unit covered by protected areas (km^2^) |  |
|  | PREPA | Presence/absence of pixels owing to protected areas |  |
| Accessibility | LROAD | Total length of roads and paths (km) | Vector layers of roads at 1:200000 spatial resolution |
|  | ACOST | Accessibility cost at 90 m spatial resolution elaborated by integrating data of slope, distance to settlements and distance to roads and paths. | Digital Elevation Models (DEM) at 90 m spatial resolution and vector layers of roads and settlements at 1:200000 spatial resolution from the Spanish Geographic Institute ([www.ign.es](http://www.ign.es)) |
