# Supplementary material for: Linking species functional traits of terrestrial vertebrates and environmental filters: A case study in temperate mountain systems
Source: PLoS One. 2019 Feb 7;14(2):e0211760. doi: 10.1371/journal.pone.0211760 (PMC6366930; doi:10.1371/journal.pone.0211760)
Supplement: S3 Table — (DOCX) [file pone.0211760.s003.docx]

**Supporting Information**

**Linking species functional traits of terrestrial vertebrates and environmental filters: a case study in temperate mountain systems**

Paula García-Llamas^1^, Thiago Fernando Rangel^2^, Leonor Calvo^1^**,** Susana Suárez-Seoane^1^

**S3 Table. CORINE Land Cover classes comprised in the Cantabrian Mountains.**

| Code | CORINE Land Cover classes |
| --- | --- |
| Human infrastructures | Continuous urban fabrics |
|  | Discontinuous urban fabrics |
|  | Industrial or commercial units |
|  | Road and rail networks and associated land |
|  | Port areas |
|  | Airports |
|  | Dump sites |
|  | Construction sites |
|  | Green urban areas |
|  | Sport and leisure facilities |
| Mineral extraction sites | Mineral extraction sites |
| Herbaceous croplands | Non-irrigated arable lands |
|  | Permanently irrigated lands |
|  | Complex cultivation patterns |
|  | Land principally occupied by agriculture, with significant areas of natural vegetation |
| Woody croplands | Vineyards |
|  | Fruit trees and berry plantations |
|  | Agro-forestry areas |
| Pasturelands | Pasturelands |
|  | Natural grasslands |
| Forest | Broad-leaved forests |
|  | Mixed forests |
|  | Coniferous forests |
| Transitional woodland-shrublands | Transitional woodland-shrub |
| Sclerophyllous-herbaceous formations | Moors and heathlands |
|  | Sclerophyllous vegetation |
| Sparsely vegetated areas | Sparsely vegetated areas |
| Bare areas | Bare rocks |
|  | Burnt areas |
| Wetlands | Peat bogs |
| Water surfaces | Inland marshes |
|  | Salt marshes |
|  | Water courses |
|  | Water bodies |
|  | Sea and ocean |
|  | Estuaries |
