# Supplementary material for: Linking species functional traits of terrestrial vertebrates and environmental filters: A case study in temperate mountain systems
Source: PLoS One. 2019 Feb 7;14(2):e0211760. doi: 10.1371/journal.pone.0211760 (PMC6366930; doi:10.1371/journal.pone.0211760)
Supplement: S4 Table — Significant predictors appear in bold. See Table 2 for codes of environmental variables. Only variables included as predictors in the most parsimonious models are shown. (DOCX) [file pone.0211760.s004.docx]

**Supporting Information**

**Linking species functional traits of terrestrial vertebrates and environmental filters: a case study in temperate mountain systems**

Paula García-Llamas^1^, Thiago Fernando Rangel^2^, Leonor Calvo^1^**,** Susana Suárez-Seoane^1^

|  |  | Feeding guild | | | | | Habitat use type | | | | | | | | | | Activity | | | *FD |
| --- | --- | --- | --- | --- | --- | --- | --- | --- | --- | --- | --- | --- | --- | --- | --- | --- | --- | --- | --- | --- |
| Family | Environmental variables | Insec. | Gran*_._* | Herb_._ | Carn_._ | Omn_._ | | Arb_._ | Anthr_._ | ***CD_._ | Gen*_._* | ***GD | RD | ***SA | ***Shru_._ | Terr_._ | ***Di_._ | Noc_._ | Mu_._ |  |
| Climate | PRECWIN | -0.18 |  | +0.01 |  |  | |  |  |  |  |  | -0.10 | -0.11 |  |  |  | **-0.14** |  |  |
|  | TMAXSUM | **+0.38** | **+0.47** |  | **+0.39** | **+0.33** | | **+0.56** | **+0.34** | **+0.26** | **+0.25** | **+0.38** | **+0.18** | **+0.35** | **+0.35** | +0.08 | **+0.01** | **+0.26** | +0.12 | **+0.017** |
| +Topography | SOLR | **+0.30** |  |  | **+0.29** | **+0.28** | | **+0.32** |  |  |  | **+0.54** | +0.08 |  | **+0.30** | +0.04 | **+0.01** |  |  |  |
|  | stdDEM |  |  | +0.13 |  |  | | **+0.37** |  |  |  |  |  |  |  |  |  |  |  |  |
|  | stdSLO | **+0.42** |  |  | **+0.47** | **+0.28** | |  | **+0.30** | **+0.23** | **+0.029** | **+0.29** | **+0.43** | **+0.22** | **+0.43** |  | **+0.004** | **+0.21** | +0.10 | **+0.041** |
| Land cover | INFRA |  |  |  |  | **-0.12** | |  |  |  |  | **-0.09** | **-0.10** | +0.05 |  |  | -0.001 |  |  |  |
|  | MIN | -0.05 |  |  | -0.05 |  | |  |  |  |  |  | -0.02 | **-0.11** |  |  |  | -0.02 | -0.06 | -3.457 |
|  | HERC |  |  | **+0.20** |  |  | |  |  |  |  | **+0.16** |  |  | **+0.27** |  |  |  |  |  |
|  | WOOC | -0.06 |  |  |  |  | | **-0.08** |  | -0.07 |  |  | -0.03 |  |  |  |  | -0.04 | -0.07 |  |
|  | PAS |  |  | +0.08 |  | **-0.11** | |  |  | **+0.14** |  | -0.06 |  |  |  |  | **-0.001** |  |  |  |
|  | FOR | **+0.15** | +0.05 | **+0.11** | **+0.12** |  | | **+0.32** | **-0.15** | **+0.12** |  |  | +0.07 |  | **+0.32** |  | **+0.001** | 0.08 | +0.03 |  |
|  | TWOOD | +0.07 |  |  | **+0.16** |  | | **+0.14** |  |  |  | **+0.14** | +0.06 | -0.08 | **+0.24** |  | **+0.001** |  |  | -0.168 |
|  | SCRUB | -0.03 | **-0.09** |  |  | **-0.11** | |  |  |  |  |  | -0.03 | -0.07 | **+0.11** | -0.05 | **-0.001** | -0.06 | -0.05 | **-0.76** |
|  | SPAR |  |  |  |  | **0.08** | |  |  | **+0.05** |  |  | +0.05 |  |  |  | **-0.001** |  | +0.03 |  |
|  | BARE |  |  |  |  |  | |  |  |  |  | **-0.09** | +0.04 |  |  |  |  |  |  |  |
|  | WET |  |  |  |  |  | |  |  | -0.07 |  | +0.06 |  |  |  | -0.02 |  |  |  |  |
|  | WAT |  |  |  |  |  | |  |  |  |  |  |  | **+0.13** |  |  |  |  |  |  |
| Physiological state of vegetation | NDVI |  |  |  |  |  | |  | **-0.11** |  |  | **-0.16** |  |  |  | **+0.21** |  | +0.07 |  |  |
| Landscape heterogeneity | *LANDHET* | **+0.09** | **+0.12** | +0.09 | +0.06 |  | | **+0.11** |  | **+0.07** |  |  | **+0.11** | +0.09 | **+0.11** | +0.02 |  | +0.02 | +0.01 | **+0.034** |
| Human influence | stdUD |  |  |  | +0.07 |  | |  | **+0.13** |  |  |  |  |  |  |  |  |  |  |  |
|  | SURFPA | **+0.31** | **+0.21** | **+0.43** | **+0.37** | **+0.28** | | **+0.37** | **+0.17** |  | **+0.16** | **+0.21** | **+.034** | **+0.28** |  | **+0.35** | **+0.004** | **+0.33** | **+0.29** | **+0.001** |
|  | PREPA |  |  | +0.07 |  |  | |  |  |  | +0.06 |  | +0.03 | **+0.11** |  | +0.05 |  | +0.06 | +0.09 | **+0.119** |
| Accessibility | LROAD |  |  | +0.08 |  | **+0.10** | |  |  |  |  |  | **+0.09** |  |  |  |  |  |  |  |
|  | ACOST | **-0.20** |  | **-0.29** | **-0.40** | **-0.24** | | **-0.32** | **-0.42** |  | **-0.43** |  | **-0.37** | **-0.32** | **-0.32** |  | **-0.002** |  | **-0.27** | **-0.083** |

**S4 Table. Results of the most parsimonious models (ordinary least squares regression and autoregresive models SAR or CAR) testing the effect of environmental predictors on the richness of each functional group, including the sign of the effect and standarized coefficient estimates of distinct predictors.** Significant predictors appear in bold. See Table 2 for codes of environmental variables. Only variables included as predictors in the most parsimonious models are shown.
